# Role of SpdA in Cell Spreading and Phagocytosis in *Dictyostelium*

**DOI:** 10.1371/journal.pone.0160376

**Published:** 2016-08-11

**Authors:** Marco Dias, Cristiana Brochetta, Anna Marchetti, Romain Bodinier, Franz Brückert, Pierre Cosson

**Affiliations:** 1 Department for Cell Physiology and Metabolism, Faculty of Medicine, Geneva University, Geneva, Switzerland; 2 Laboratoire des Matériaux et du Génie Physique (LMGP), UMR CNRS-Grenoble INP5628 Université Grenoble Alpes, 3 parvis Louis Néel, BP 257, Grenoble cedex 1, France; MRC Laboratory of Molecular Biology, UNITED KINGDOM

## Abstract

*Dictyostelium discoideum* is a widely used model to study molecular mechanisms controlling cell adhesion, cell spreading on a surface, and phagocytosis. In this study we isolated and characterize a new mutant created by insertion of a mutagenic vector in the heretofore uncharacterized *spdA* gene. *SpdA-ins* mutant cells produce an altered, slightly shortened version of the SpdA protein. They spread more efficiently than WT cells when allowed to adhere to a glass substrate, and phagocytose particles more efficiently. On the contrary, a functional *spdA* knockout mutant where a large segment of the gene was deleted phagocytosed less efficiently and spread less efficiently on a substrate. These phenotypes were highly dependent on the cellular density, and were most visible at high cell densities, where secreted quorum-sensing factors inhibiting cell motility, spreading and phagocytosis are most active. These results identify the involvement of SpdA in the control of cell spreading and phagocytosis. The underlying molecular mechanisms, as well as the exact link between SpdA and cell spreading, remain to be established.

## Introduction

Phagocytosis is the process by which eukaryotic cells ingest big particles (diameter typically >1μm) such as bacteria or cell debris. This process plays a key role in the defense of mammalian organisms against invading microorganisms [1], as well as in the clearance of dead cells continuously generated by cell division and apoptosis [2]. Phagocytosis is a complex process that is initiated by binding of a phagocytic cell to a particle. This initial binding triggers a local activation cascade, leading to a local reorganization of the actin cytoskeleton and a change in cell shape, ultimately allowing the engulfment of the particle into a closed phagosome [3]. Cellular adhesion, regulated dynamics of the actin cytoskeleton, membrane fusion and fission events are at play at multiple steps of the phagocytic process, and multiple molecular players are implicated in this process. In addition, many signaling pathways regulate this core adhesion machinery and control cellular adhesion and particle engulfment [3].

*Dictyostelium discoideum* is a widely used model to study phagocytosis. This social amoeba lives in the soil, where it feeds by continuously ingesting microorganisms. The molecular processes at play are similar to those characterized in mammalian cells, and implicate notably an adhesion molecule with integrin beta features, which interacts with talin and myosin VII to regulate actin dynamics [4]. Many other gene products participating in phagocytosis directly or indirectly (for example by controlling surface levels of adhesion molecules) have been characterized in this system. One of the key experimental advantages of *Dictyostelium discoideum* is its small haploid genome, allowing the relatively easy generation of random and targeted mutant cells. Typically, the role and relative importance of any given gene product in phagocytosis is determined in this organism by comparing the rate of phagocytosis of the corresponding KO strain with that of parental cells. Some mutations can reduce phagocytosis efficiency drastically [5], others show more modest defects [6], and a few were reported to increase phagocytosis [7–9]. Upon detailed analysis, each gene product can usually be classified as directly involved in adhesion to phagocytic particles or in engulfment, or as a regulator of the phagocytic process.

In this study we report the identification and characterization of a new gene product, named *spdA*, which is involved in cell spreading. Genetic alterations of *spdA* modify the ability of cells to spread on a substrate, and to phagocytose particles.

## Materials and Methods

### Isolation of *spdA-ins* mutant cells

All *Dictyostelium* strains used in this study were derived from the subclone DH1-10 [5] of the DH1 strain, referred to as wild-type (WT) for simplicity. Cells were grown at 21°C in HL5 medium (14.3 g/L peptone (Oxoid, Hampshire, England), 7.15 g/L yeast extract, 18 g/L maltose monohydrate, 0.641 g/L Na_2_HPO_4_^.^2H_2_O, 0.490 g/L KH_2_PO_4_) and subcultured twice a week to maintain a maximal density of 10^6^ cells/ml. Unless otherwise specified, all experiments presented in this study were done with cells grown to a density of approximately 500’000 cells per mL.

**Fig 1:**
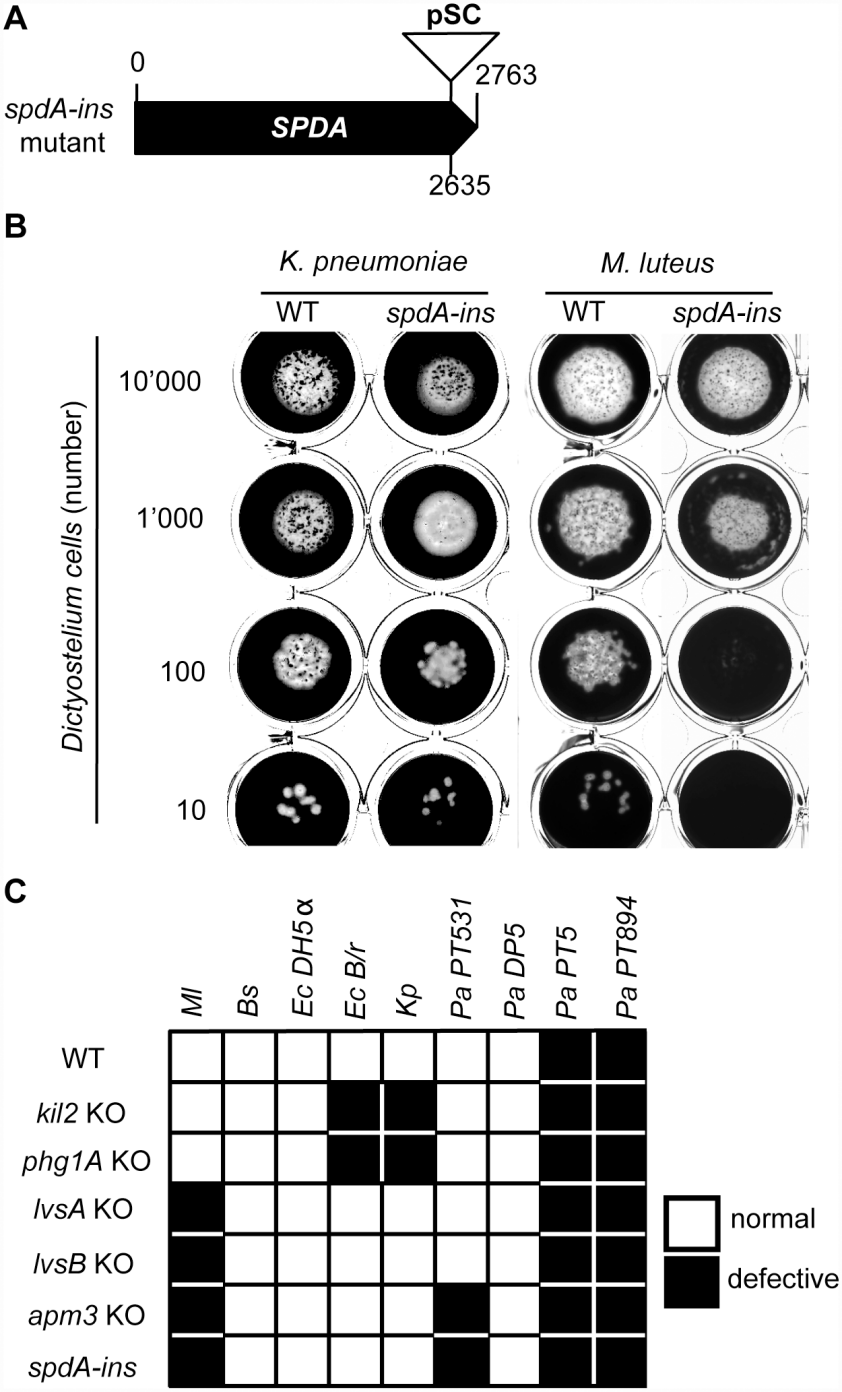
Characterization of *spdA-ins* mutant cells. (A) *SpdA-ins* mutant cells were originally created by the random insertion of a REMI mutagenic vector (pSC) in the coding sequence of gene DDB_G0287845 (position 2635). (B) To quantify growth of *Dictyostelium* on bacteria, we applied 10'000, 1'000, 100 or 10 *Dictyostelium* cells on a lawn of *K*. *pneumoniae* or *M*. *luteus* bacteria (black). WT cells created a phagocytic plaque (white). *SpdA* mutant cells grew as efficiently as WT cells on a lawn of *K*. *pneumoniae* but less efficiently in the presence of *M*. *luteus*. (C) Growth of *Dictyostelium* mutant strains in the presence of different bacterial species.

Random mutants were generated by restriction enzyme-mediated integration (REMI) mutagenesis [10], subcloned individually, then tested for their ability to grow on a lawn of bacteria as described previously [11]. In this study, one mutant growing inefficiently on a laboratory strain of *Micrococcus luteus* (*Ml*) bacteria was selected for further analysis. The genomic DNA from these *spdA-ins* mutant cells was recovered, digested with ClaI and religated, and the mutagenic plasmid was recovered together with the flanking regions of its genomic insertion site (Fig 1). This plasmid was sequenced to identify the insertion site. It was also used after ClaI digestion to transfect WT cells and generate targeted *spdA-ins* mutant cells. Three independent *spdA-ins* mutant clones were generated, and used in parallel in this study, with indistinguishable results (Fig 1).

### Phagocytosis and Fluid Phase Uptake

Phagocytosis and fluid phase uptake were determined as described previously [5] by incubating cells for 20 min in suspension in HL5 medium containing either 1-μm-diameter Fluoresbrite YG carboxylate microspheres (Polysciences, Warrington, PA) or Alexa647-dextran (Life Technologies, Eugene, OR). Cells were then washed twice with HL5 containing 0.2% NaN_3_, and the internalized fluorescence was measured by flow cytometry [5]. Kinetics of phagocytosis were determined similarly after 0, 5, 10, 15, 20, 30, 40, 60, 90, 120 and 150 min of incubation. Since these experiments required a large number of cells, the cells were grown to a higher concentration than for all other experiments described in this study (1.5x10^6^ cells per mL vs 500’000 cells per mL). This accounts for the fact that phagocytosis was less efficient for all strains in this set of experiments.

To determine if the elevated phagocytosis observed in *spdA-ins* mutant cells was a cell-autonomous phenotype, we mixed WT cells and *spdA-ins* mutant cells expressing GFP (ratio 1:1). After three days of co-culture in HL5, phagocytosis was analyzed as described above, but using latex beads fluorescent in the rhodamine channel (Polysciences, Inc). During flow cytometry, GFP fluorescence allowed to distinguish WT from *spdA-ins* mutant/GFP cells.

### Cell spreading and motility

In order to visualize cell spreading on a substrate, 1.5x10^5^ cells were allowed to adhere for 20 min on a glass surface in a FluoroDish (World Precision Instr., Sarasota, FL). To monitor the presence and spreading of *D*. *discoideum* cells we used an inverted microscope (Olympus IX71 or Zeiss Axiovert 100M) and imaged by phase contrast and Reflection Interference Contrast Microscopy (RICM) as previously described [12]. Images and movies (15 frames per second) were acquired with an Olympus DP30 CCD camera or a High resolution black/white CCD camera (Hamamatsu CCD cooled camera). RICM images were sub-sampled at 1 image per 1.2 s, the background was subtracted and flattened and the noise filtered. Dark cell-surface contact zones were defined by segmentation and quantified as described [13].

To measure cell motility, cells were observed for 60 min (picture every 30 sec) by phase contrast with a Plan-Neofluar 10x magnification. Pictures were taken with a Hamamatsu CCD cooled camera. We used Particle tracking from the Metamorph software to track individual cell trajectories.

### Cell detachment assays

In these experiments, borosilicate glass plates were first cleaned with detergent in alkaline conditions, then briefly detached with a 14 M NaOH solution, thoroughly rinsed and dried. Radial flow experiments were performed essentially as described previously [14, 15]. Briefly, cells were resuspended in HL5 (10^6^ cells/mL) and allowed to attach on the glass surface during 10 min, then a radial hydrodynamic flow was applied for 10 min and the density of the remaining attached cells was determined as a function of the distance to the center of the flow. The results were expressed as the percentage of detached cells as a function of the applied shear stress.

### Microscopy

To visualize filamentous actin, cells were allowed to adhere on a glass coverslip for 10 min in HL5 and were fixed in PB containing 4% paraformaldehyde for 30 min. This fixation was sufficient to permeabilize the cells. The actin cytoskeleton was labeled by incubating the cells for 1h in phosphate-buffered saline (PBS) containing 0.2% bovine serum albumin and 1μg/ml tetramethylrhodamine B isothiocyanate (TRITC)-labeled phalloidin. The coverslips were then washed twice, mounted and observed by laser scanning confocal microscopy (Zeiss LSM 510). The pictures presented represent optical sections at the site of contact between the cells and the substrate.

For scanning electron microscopy, cells were incubated on glass coverslips overnight in HL5. Cells were then fixed with 2% glutaraldehyde in HL5 for 30 min followed by 2% glutaraldehyde in 100 mM PB (pH 7.14) for 30 min. Cells were rinsed and postfixed in 1% osmium tetroxide in 100 mM PB (pH 7.14) for 1 h. The fixative was removed, and cells were progressively dehydrated through a 25–100% ethanol series. After air-drying, cells were sputter-coated in gold and viewed on a JEOL-JSM-7001 FA Field Emission Scanning Electron Microscope.

### Measuring cell size

To measure cell size based on electric current exclusion (CASY technology), cells were grown to a density of 50x10^4^ cells/mL, diluted to 1x10^4^ cells/mL, and 10 mL were analyzed using a CASY 1 cell counter (Roche; CASY Model TTC).

To determine packed cell volume, cells are grown to a density of 3x10^6^ cells/mL. Cells were counted under a Nikon eclipse TS100 microscope with a cell counting Neubauer chamber. 3x10^6^ cells in 1 mL were transferred in the packed cell volume (PCV) tube with calibrated capillary and volume graduation (5μL) (TPP Techno Plastic Products AG; Product no 87005). Cells were centrifuged 2 min at 1500 rpm, and the pellet volume measured in the calibrated capillary. The ratio μL of pellet/number of cells was calculated.

To measure the amount of protein per cell, 10^6^ cells were collected by centrifugation, washed once in 1ml PBS, resuspended in 50 μL PBS containing triton X-100, 0.05% and transferred to a 96 well plate. To quantify protein content using a Lowry assay (DC Protein Assay, Bio-RAD) we added to each well 25 μL of reagent A and 200μL reagent B. After 15 min the absorbance at 750 nm was measured in a microplate reader, and compared to a set of calibrated serial dilutions.

### Western blot analysis

To determine the cellular amounts of SibA, Phg1 and Talin, we resuspended cell pellets in sample buffer and separated the proteins on a polyacrylamide gel (7% for SibA and Talin and 10% for Phg1), after which they were transferred to a nitrocellulose membrane (Invitrogen, Carlsbad, CA). The membranes were incubated with a polyclonal anti-SibA antibody (SibA) [12], the YC1 rabbit antipeptide antiserum to a Phg1 peptide [5], or the anti-talin 169.477.5 [16], a kind gift from Prof. G. Gerisch (Martinsried, Germany). Binding of antibodies was revealed by ECL using a secondary HRP-coupled antibody (Amersham Biosciences). The signal intensity was evaluated using Quantity One software (Bio-Rad).

## Results

### Selection of *spdA-ins* mutant cells

Mutations affecting the organization or function of the endocytic/phagocytic pathway were previously shown to alter the ability of *Dictyostelium* cells to feed upon bacteria, while preserving their ability to feed upon HL5 medium. In order to identify new gene products potentially involved in phagocytosis, we created a library of random mutants by Restriction Enzyme Mediated Insertion, as described previously [11]. Briefly, a BamHI-digested pSC vector and the SauIIIA restriction enzyme were introduced in cells by electroporation. After selection of stably transfected cells in the presence of blasticidin, individual mutant cells were cloned by flow cytometry, and their ability to grow upon a variety of bacteria was tested. Previous results suggested that different gene products are essential for efficient growth on *K*. *pneumoniae*, *P*. *aeruginosa*, *M*. *luteus*, and *B*. *subtilis*, and that their common denominator is to be linked to one of the facets of the phagocytic process (adhesion, phagocytosis, intracellular killing…) [11]. The *spdA-ins* mutant was initially isolated as a mutant growing poorly on a lawn of *M*. *luteus*, a gram-positive bacterium. The mutagenic pSC plasmid was recovered from *spdA-ins* mutant cells, and the flanking genomic regions were sequenced. In *spdA-ins* mutant cells, the pSC plasmid is inserted on chromosome 5, 2636 nucleotides downstream of the start codon of DDB_G0287845 (Figs 1A and S1), hereafter called *spdA*. New *spdA-ins* mutant cells were generated by homologous recombination and three independent mutant lines were used in parallel for further characterization (S1 Fig). The predicted SpdA protein is composed of 920 amino acids, and the insertion of pSC in the coding sequence of *SPDA* results in the production of a truncated protein where the last 41 aminoacid residues are deleted. The SpdA protein exhibits no transmembrane domain or previously characterized domains. No clear ortholog could be identified in mammals or other species. A region extending from positions 3 to 450 shows homology with the N-terminal portion of many proteins from *D*. *discoideum*, *D*. *purpureum* and *D*. *lacteum* and may represent a novel undescribed domain. In *Dictyostelium discoideum*, the most conserved region encompassed the first 120 residues, and a Genome Blast search identified 18 gene products with homology to the N-terminal region of SpdA (S1 Table). Besides a conserved N-terminal region, none of these gene products exhibits a known functional domain. We are proposing to name the members of this putative new family SpdA to SpdS (S1 Table).

Growth of *spdA-ins* mutant cells in HL5 medium was unaffected compared to WT cells. To quantify growth of *Dictyostelium discoideum* strains on bacteria, 10'000, 1’000, 100 or 10 *Dictyostelium* cells were applied onto a bacterial lawn (Fig 1B). After 5 days in the presence of *K*. *pneumoniae* bacteria or of several other bacterial species, WT and *spdA-*ins mutant cells created phagocytic plaques cleared of bacteria with similar efficiencies (Fig 1B and 1C). On the contrary *spdA-ins* mutant cells grew less efficiently on a lawn of *M*. *luteus* and on the non-virulent PT531 *P*. *aeruginosa* strain (Fig 1B and 1C). Other mutant cells with poor growth on *M*. *luteus* characterized in our laboratory include *lvsA* KO cells [6], *lvsB* KO cells [6] and *apm3* KO cells [17] (Fig 1C). Since these three mutants have shown alterations in the organization and function of the endocytic and phagocytic pathway, we next tested the ability of *spdA-ins* mutant cells to perform phagocytosis and endocytosis.

### *SpdA-ins* mutant cells phagocytose particles more efficiently than WT cells

**Fig 2:**
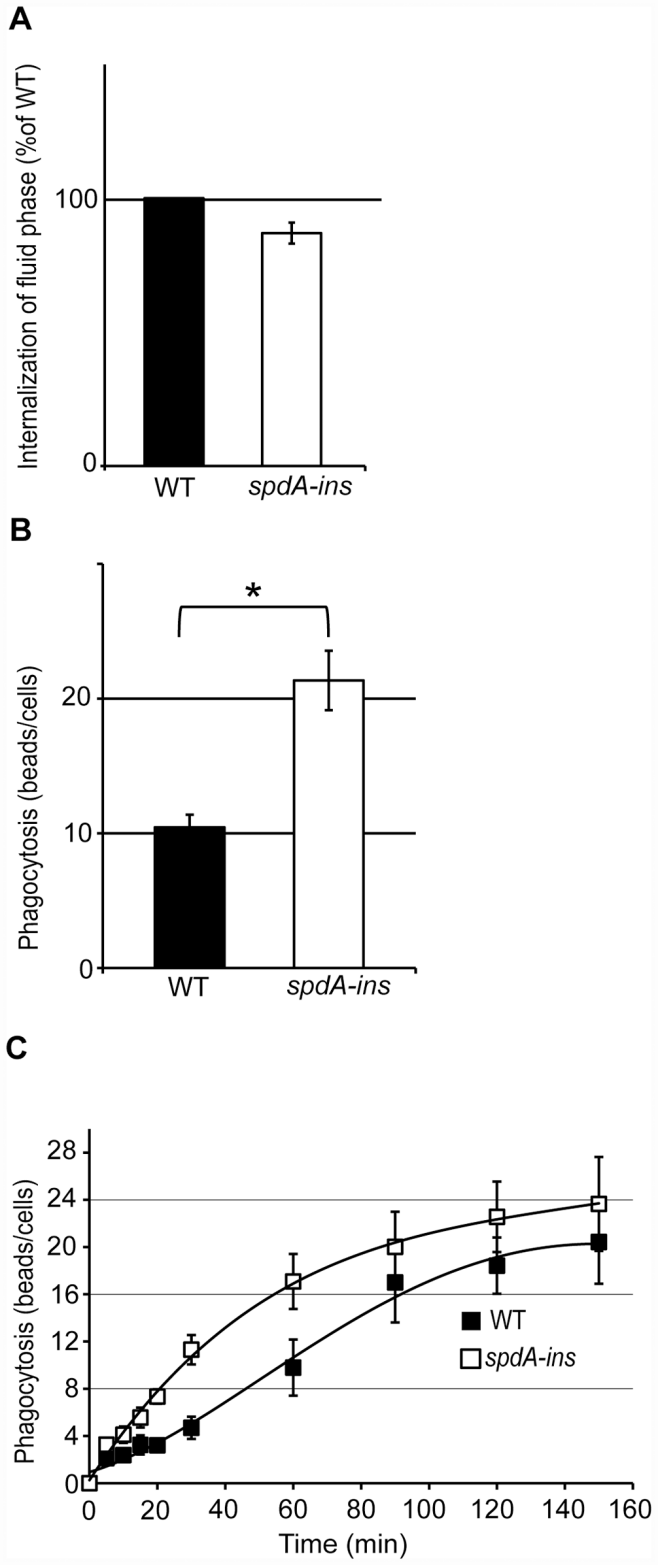
S*pdA-ins* mutant cells phagocytose particles faster than WT cells. Cells were incubated for 20 min in HL5 medium containing fluorescent dextran or fluorescent latex beads. Cells were then washed, and internalized fluorescence was measured by flow cytometry. (A) Uptake of fluorescent dextran was expressed as a percentage of the value obtained for the WT cells. (B) Phagocytosis of fluorescent beads was expressed as the average number of beads ingested per cell. The average and SEM of 6 independent samples are presented. *: p<0.01 (Student t-test). (C) Cells were incubated for 0, 5, 10, 15, 20, 30, 60, 90, 120, or 150 min in HL5 medium containing fluorescent latex beads. The average and SEM of 4 independent experiments are presented. *SpdA-ins* mutant cells ingested particles faster than WT cells.

To assess the function of the endocytic and phagocytic pathways, *spdA-ins* mutant cells and WT cells were incubated for 20 min in the presence of fluorescent dextran or fluorescent latex beads (1μm diameter). The internalized material was then measured by flow cytometry. Macropinocytosis of fluid phase was similar in WT cells and in *spdA-ins* mutant cells (Fig 2A). On the contrary, *spdA-ins* mutant cells phagocytosed latex beads more efficiently than WT cells (Fig 2B).

Analysis of phagocytosis kinetics further revealed that this difference was due to an increased rate of phagocytosis, evident at early phagocytosis times, while accumulation of ingested beads reached a similar plateau in WT and *spdA-ins* mutant cells after 120 min (Fig 2C). Phagocytosis and macropinocytosis both rely on similar rearrangements of the actin cytoskeleton, while phagocytosis requires in addition efficient adhesion of the cell to particles. Consequently several mutants defective in cell adhesion have been found to exhibit a decreased phagocytosis and no decrease in macropinocytosis [5, 12, 18].

**Fig 3:**
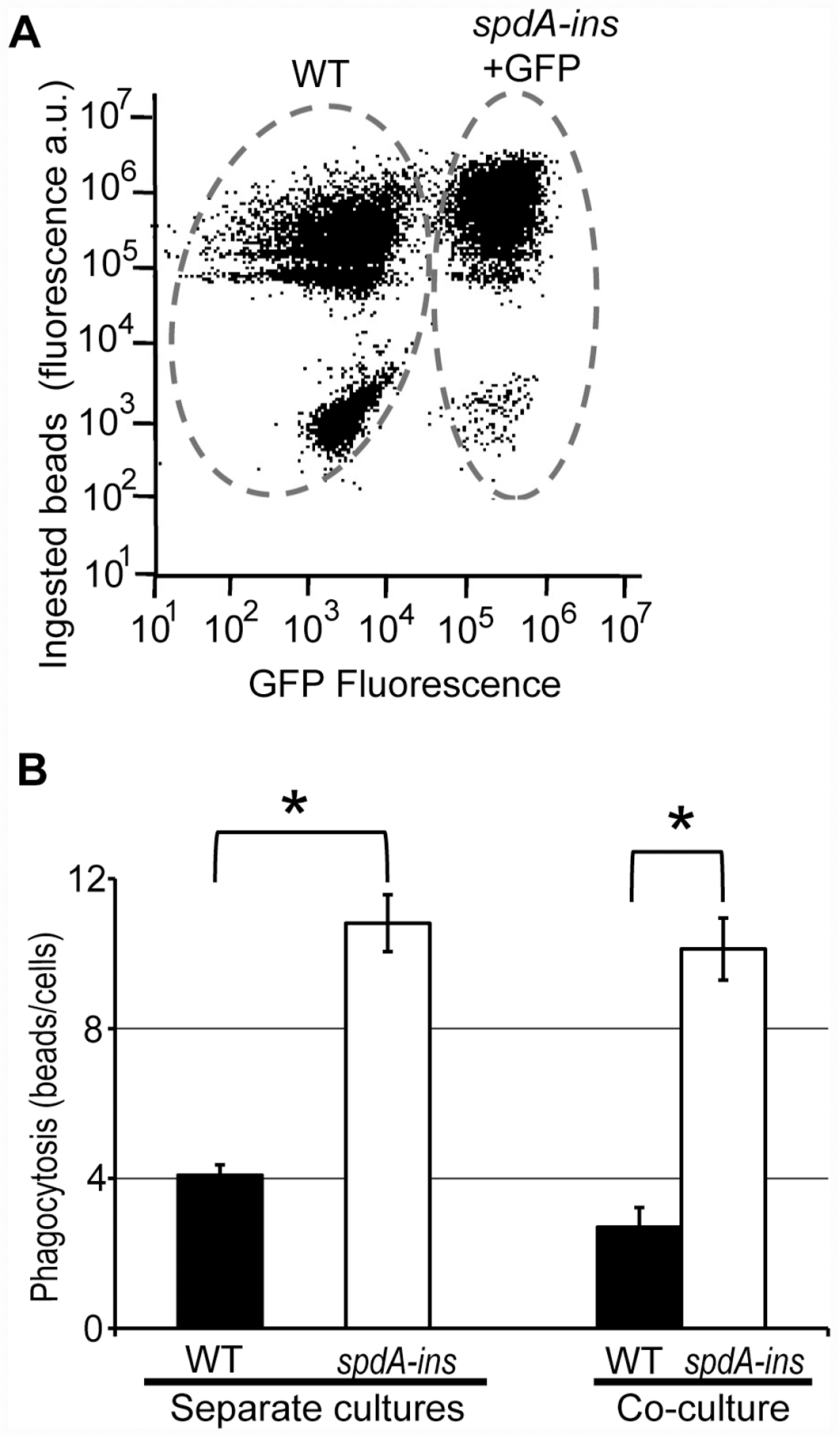
The phenotype of *spdA-ins* mutant cells is cell autonomous. (A) *SpdA* mutant cells expressing GFP were mixed with WT cells and cultured for three days. We then incubated the cells with rhodamine-labeled latex beads and assessed phagocytosis by flow cytometry. Expression of GFP allowed to distinguish WT cells from *spdA* mutant cells, and revealed that *spdA* mutant cells co-cultured with WT cells phagocytosed more efficiently than WT cells. (B) The phagocytosis of WT and *spdA-ins* cells cultured separately or co-cultured is indicated (mean±SEM; n = 6). *: p<0.01 (Student t-test).

To determine if the phenotype of *spdA-ins* mutant cells was cell-autonomous, we mixed *spdA-ins* mutant cells expressing GFP with WT cells. After three days of co-culture, we assessed the phagocytosis of rhodamine-labeled latex beads by flow cytometry. Expression of GFP allowed the identification of mutant cells (Fig 3A), and revealed that *spdA-ins* mutant cells co-cultured with WT cells still phagocytosed more efficiently than WT cells (Fig 3B).

### S*pdA-ins* mutant cells adhere better than WT cells to their substrate

**Fig 4:**
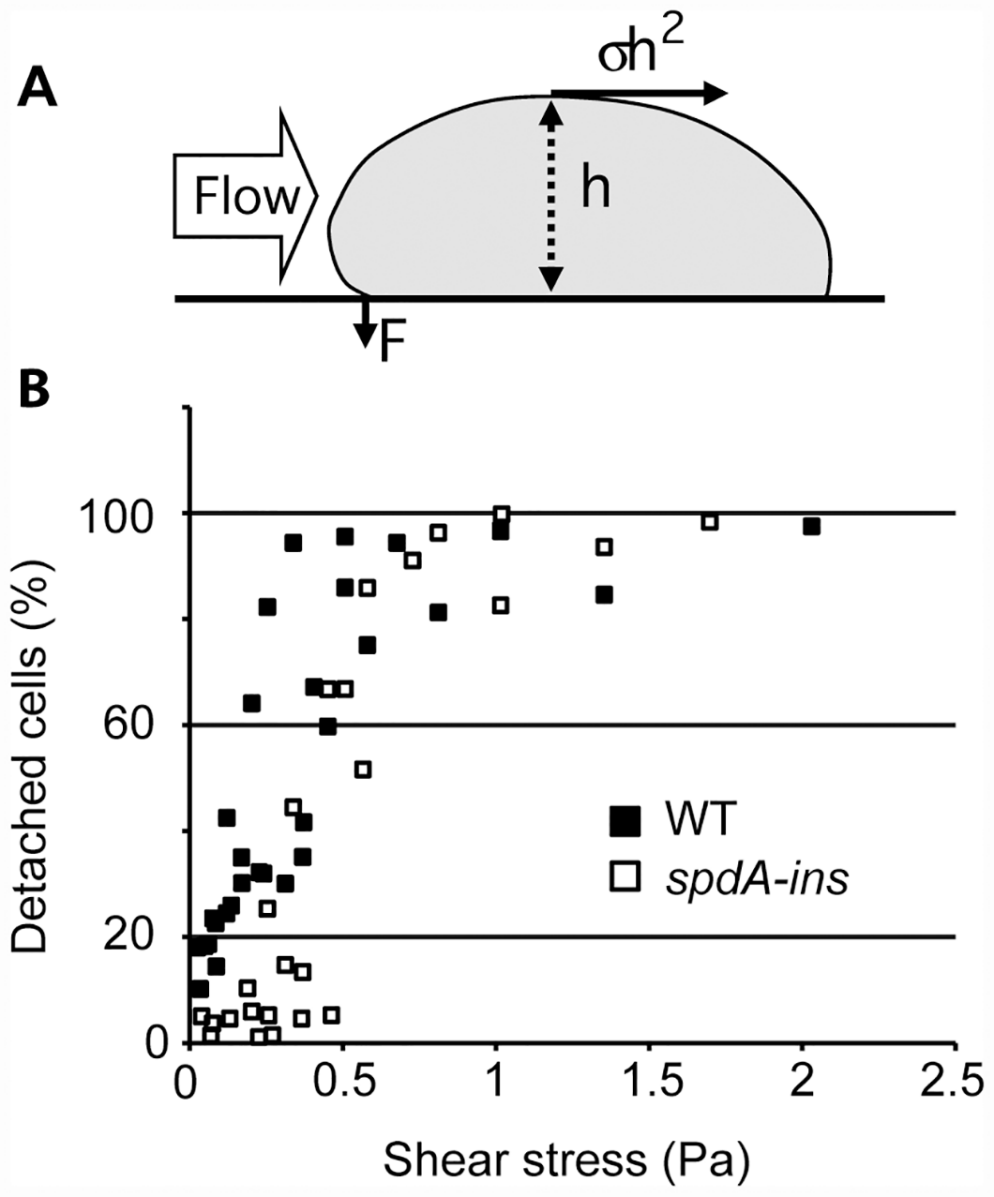
*SpdA-ins* cells adhere more efficiently than WT cells to their substrate. (A) Side view of a cell attached to its substrate and exposed to a flow of medium. The adhesion of the cell to its substrate can be assessed by determining the speed of a flow of medium that is necessary to detach the cells [19]. The strength applied by the flow of medium on the cell is σh^2^, and its mechanical moment (σh^3^) is balanced by the adhesive force (F). Inspired from [18]. (B) Percentage of detached cells as a function of the applied shear stress. At a low flow (between 0 and 0.5 Pa), *spdA-ins* cells detached less readily than WT cells from the substrate. At higher flow (>0.5 Pa) no significant difference can be seen between WT cells and *spdA-ins* cells. Data from three independent experiments is represented in this graph. A decrease in cell detachment can be the result of an increase in the adhesion force (F) or of a decrease in h (i.e. of a more efficient cell spreading).

In order to assess the efficiency with which cells adhered to a substrate, cells were allowed to attach to a glass substrate, then subjected to a radial hydrodynamic flow for 10 min (Fig 4A) [19]. The shear stress applied to the cells depends on the velocity of the flux, which decreases when the distance to the center of the flow increases (Fig 4A). The percentage of cells detached by the flow was determined as a function of the distance to the center, and the results expressed as the percentage of detached cells as a function of the applied shear stress. At a low shear stress, between 0 and 0.5 Pa, *spdA-ins* mutant cells detached less readily than WT cells from the substrate (Fig 4B). When the shear stress was higher than 0.5 Pa, almost 100% of both WT and *spdA-ins* mutant cells detached from the substrate (Fig 4B). These results indicate that *spdA-ins* mutant cells adhered more efficiently than WT cells to their substrate. An alternative interpretation is that mutant cells spread more readily on their substrate, which would decrease cell height (h in Fig 4A) and thus reduce the shear stress.

### *SpdA-ins* mutant cells spread more efficiently than WT cells on a substrate

**Fig 5:**
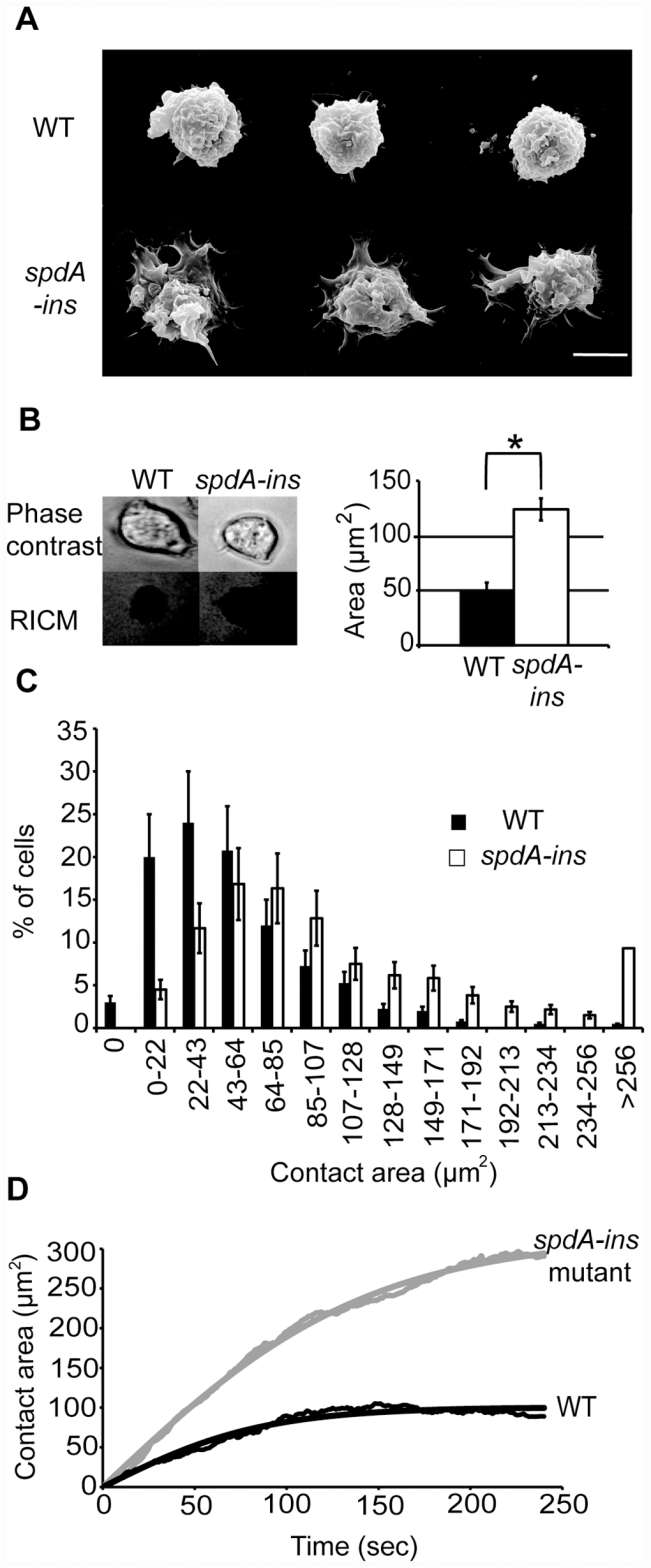
*SpdA-ins* cells spread more efficiently than WT cells. (A) WT and *spdA-ins* cells attached to a glass substrate were examined by scanning electron microscopy. Three representative pictures of each cell line are shown. *SpdA* cells spread more efficiently on their substrate than WT cells. Scalebar: 10μm. (B) To quantify cell spreading, cells were examined by phase contrast and RICM (Reflexion Interference Contrast Microscopy). The contact area between cells and their substrate appears black and it was quantified for WT and *spdA-ins* cells (mean±SEM; n = 4 independent experiments. In each experiment 20 cells were analysed for each sample). *: p<0.01(Student t-test). (C) The contact area of individual cells is represented for the whole population of cells analyzed. (D) Kinetics of cell spreading was determined. S*pdA-ins* cells spread more and faster than WT cells. Thin lines: average spreading kinetics of cells (11 and 6 cells, respectively, representative of 3 independent experiments). Solid lines: fit with eq. 7 in ref 13, A(t) = A_max_ tanh (αt).

The spreading of cells on a substrate was first visualized by Scanning Electron Microscopy. WT cells appeared round and they did not spread much on their substrate. On the contrary, *spdA-ins* mutant cells formed extended contacts with the substrate, and their height may be reduced (Fig 5A). To quantify the difference between these two phenotypes, we visualized by Reflection Interference Contrast Microscopy (RICM) the size of the contact area between cells and their substrate. Cells were allowed to adhere to their substrate for 10 minutes, then they were observed by phase contrast and by RICM (Fig 5B). The average contact area between cells and their substrate (black in RICM) was quantified, and was significantly higher for *spdA-ins* mutant cells than for WT cells (Fig 5B). When the contact area of each cell was plotted (Fig 5C) the whole population of *spdA-ins* mutant cells presented an increased area of close contact with the glass substrate. Finally, we measured the kinetics of cell spreading by RICM, and observed that *Dictyostelium spdA-ins* mutant cells spread more and faster than WT cells (Fig 5D). These observations confirm the proposal that *spdA-ins* mutant cells spread more efficiently on their substratum.

**Fig 6:**
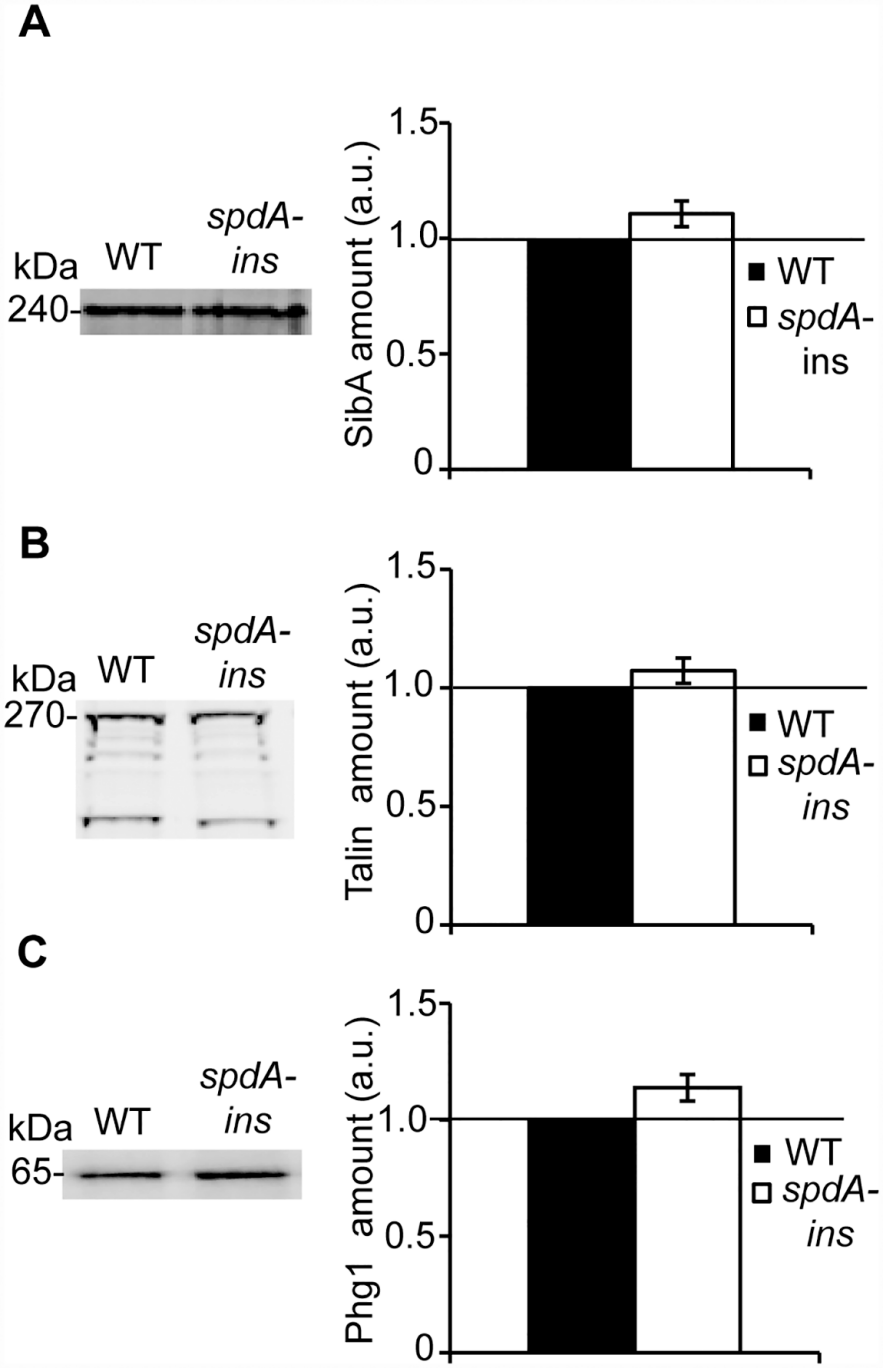
The cellular amounts of SibA, Phg1 and Talin are similar in WT cells and in *spdA-ins* cells. To determine the cellular amount of SibA, Pgh1A or Talin, cellular proteins were separated by electrophoresis and specific proteins revealed with antibodies against SibA (A), Talin (B) or Phg1A (C). The intensity of the signal was quantified and expressed in arbitrary units (a.u.). The average and SEM of four independent experiments are represented. The amounts of SibA, Phg1a and Talin were similar in WT cells and in *spdA-ins* cells.

An increased spreading could result from increased expression of cellular proteins involved in cell adhesion, such as Talin [20], SibA [12] or Phg1A [5]. In order to evaluate this possibility, we analyzed by Western blot the amount of these proteins in WT and *spdA-ins* mutant cells. The amounts of SibA, Talin and Phg1A appeared very similar in WT and *spdA-ins* mutant cells (Fig 6).

**Fig 7:**
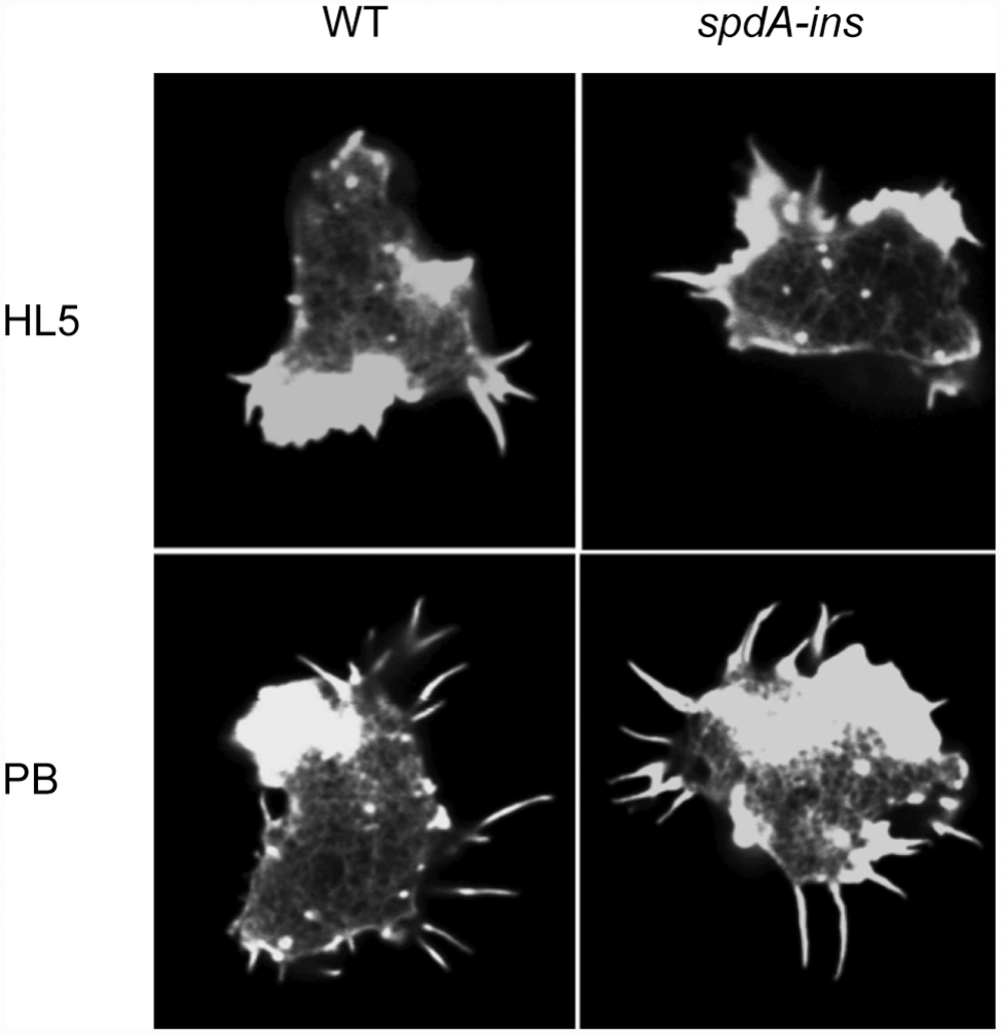
The actin organization is not significantly altered in *spdA-ins* cells. Cells were allowed to adhere to a glass coverslip for 10 min in HL5. After fixation filamentous actin was labeled with fluorescent phalloidin. The contact area between cells and their substrate was visualized by confocal microscopy, and did not reveal gross alterations of actin organization in *spdA-ins* cells. When cells were incubated in phosphate buffer (PB), formation of filopodia was induced in both WT and *spdA-ins* cells.

We also visualized the organization of the actin cytoskeleton in WT and *spdA-ins* cells by fluorescence microscopy. For this, cells were allowed to adhere to a glass coverslip, fixed and stained with fluorescent phalloidin and observed by confocal microscopy. Pictures were taken in the region where cells are in contact with the substratum. The organization of actin did not appear significantly different in WT cells and in *spdA-ins* mutant cells showing small actin foci and peripheral accumulation at sites where pseudopods emanate from the cell body (Fig 7). Incubation in phosphate buffer induces the formation of filopodia in WT *Dictyostelium* cells [21] and a similar phenotype was observed in *spdA-ins* mutant cells (Fig 7). Similarly, the random migration of cells, a phenomenon highly dependent on the dynamics of the actin cytoskeleton was similar in WT and *spdA-ins* mutant cells (1.5 μm/min) (S2 Fig). Thus, the increased ability of *spdA-ins* mutant cells to spread on a substratum was not associated with a gross alteration of the organization or dynamics of the actin cytoskeleton.

### The size of WT and *spdA-ins* mutant cells is similar

**Fig 8:**
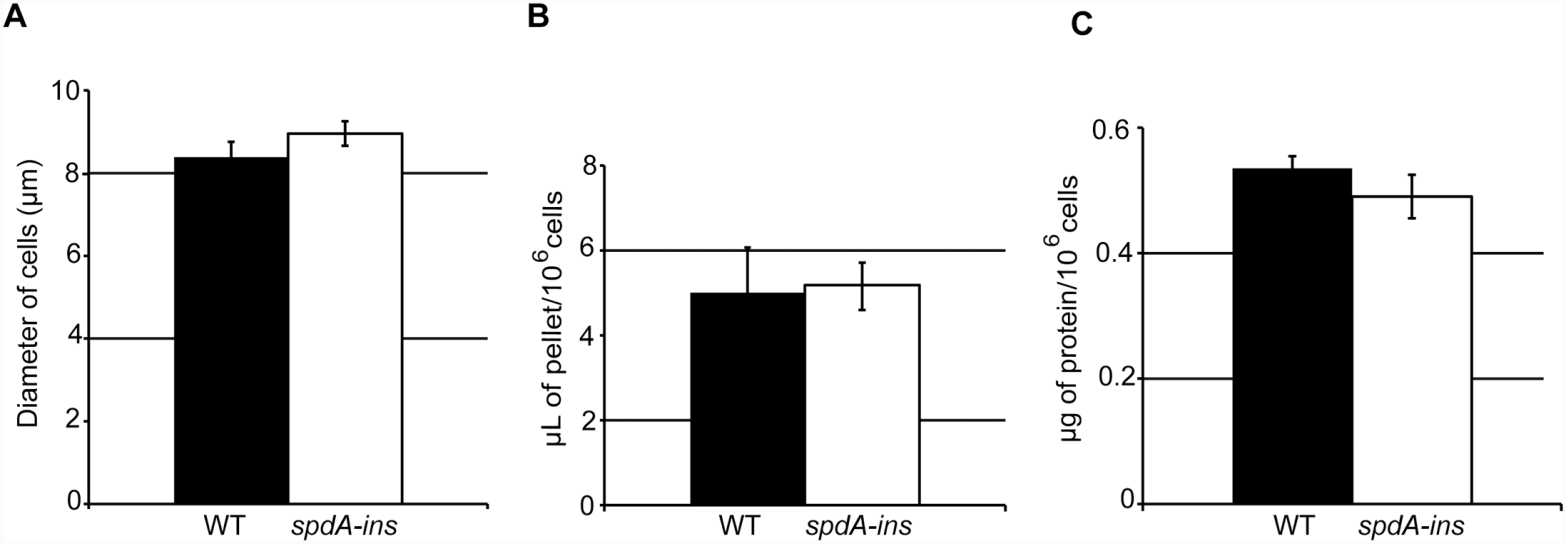
WT and *spdA-ins* cells have similar sizes. (A) Cell size was analyzed by electric current exclusion using a CASY 1 cell counter. (B) The packed cell volume of a known number of cells was determined in graded tubes. (C) The amount of protein per cell was determined using a Lowry assay. For each experiment, the average and SEM of three independent experiments is indicated. No significant differences were seen between WT and *spdA-ins* cells.

An increase in phagocytosis or in the area of contact with the substrate could potentially result from an increase in cell size, all other parameters unchanged. We used three independent methods to measure the size of WT and mutant cells. First, we used flow cytometry associated with a measure of electric current exclusion (CASY technology). The diameter of WT and of *spdA-ins* mutant cells was very similar (respectively 8.4 μm and 8.95 μm) (Fig 8A). Second, the packed cell volume was measured using graded centrifugation tubes, and was highly similar for WT and *spdA-ins* mutant cells (Fig 8B). Third, the amount of protein per cell was similar in WT and *spdA-ins* mutant cells (Fig 8C). Together these experiments indicate that WT and *spdA-ins* mutant cells have similar sizes.

### Phagocytosis and spreading are decreased in *spdA* KO cells

In *spdA-ins* mutant cells the mutagenic plasmid was inserted close to the 3’ of the *spdA* coding sequence resulting in the production of a slightly shorter version of the SpdA protein (See Fig 1A). This aberrant protein may either be functionally inactive or unstable, or on the contrary exhibit an increased activity or stability. To distinguish between these two possibilities, we generated by homologous recombination *spdA* KO cell lines where a large part of the *spdA* coding sequence was deleted (S3 Fig). In three independent clones of *spdA* KO cells, phagocytosis was found to be reduced compared to WT cells (51±4% of WT phagocytosis; mean±SEM; n = 6), while internalization of fluid phase was unchanged (103±2% of WT uptake; mean±SEM; n = 6). In addition, the spreading of *spdA* KO cells on a substrate was less efficient than that of WT cells (83±2% of WT spreading; mean±SEM; n = 7). This did not reflect a change in the size of the *spdA* KO cells, which was unchanged compared to WT cells (diameter WT 8.90±0.1μm; *spdA* KO 9.0±0.1μm; n = 6).

The fact that the effect of a spdA genetic inactivation is to decrease phagocytosis and cell spreading confirms the implication of SpdA in these cellular functions. Moreover it indicates that SpdA either participates directly in cell spreading, or acts as an activator of these functions.

**Fig 9:**
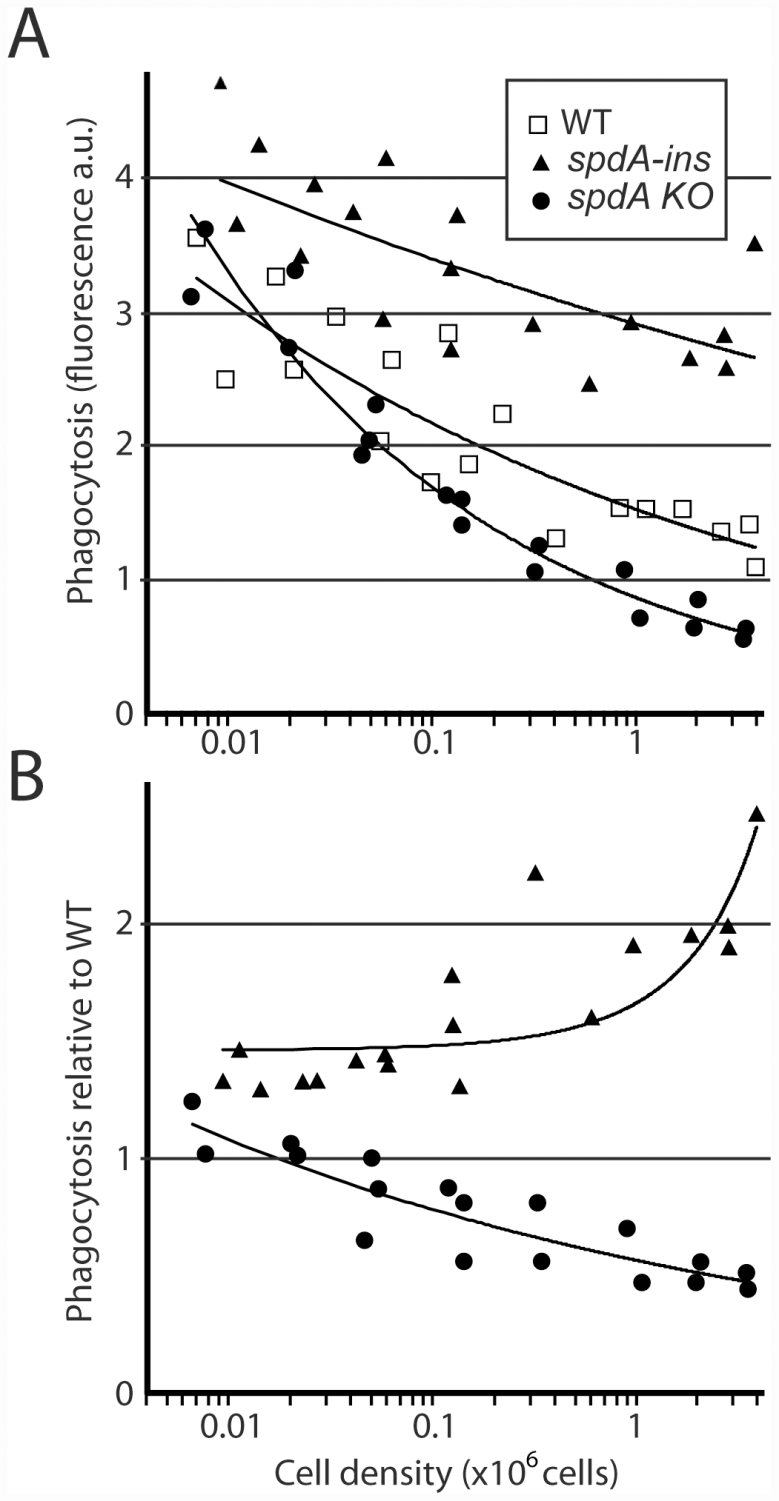
Role of SpdA in the regulation of phagocytosis by cell density. (A) Cells were grown to the indicated densities, and allowed to phagocytose fluorescent latex beads for 20 minutes. Phagocytosis was measured by flow cytometry. The results of three independent experiments were pooled in this figure. (B) In the experiment described in A, phagocytosis in mutant cells was directly compared to phagocytosis by WT cells grown at the same density. While both mutant cells phagocytosed like WT cells at low cell density, marked differences appeared when cellular density increased.

Cell adhesion and phagocytosis are heavily regulated processes in Dictyostelium, and are in particular sensitive to cell density [22]. In order to evaluate if SpdA may participate in the regulation of cell spreading and phagocytosis, we grew cells at different densities, and assessed their ability to phagocytose latex beads. In WT *Dictyostelium* cells, phagocytosis strongly decreased when cellular density increased (Fig 9A). At very low cell density, phagocytosis was almost as efficient in WT, *spdA-ins* mutant cells, and *spdA KO* cells. On the contrary, as cell density increased, phagocytosis decreased less in *spdA-ins* mutant cells than in WT cells, and more in *spdA KO* cells than in WT cells (Fig 9).

## Discussion

In this study we identified a new gene implicated in the control of cell adhesion and spreading, named *spdA*. *SpdA-ins* mutant cells spread faster and more efficiently than WT cells. They also phagocytose particles more efficiently than WT cells. These mutant cells were originally isolated based on the observation that they were defective for growth in the presence of *M*. *luteus* bacteria. It is likely that the inability of *spdA-ins* mutant cells to grow in the presence of *M*. *luteus* reflects the observed alterations in the function of the phagocytic pathway. This observation confirms that isolation of mutants defective for growth on certain bacteria does allow to identify new genes implicated in the organization and function of the endocytic and phagocytic pathways.

Previous studies have suggested that cell adhesion, phagocytosis and cell motility can be modulated negatively by secreted quorum-sensing factors in *Dictyostelium* [22, 23]. This allows cells to modulate their behavior as a function of cell density. Strikingly, at very low cell densities, both *spdA-ins* and *spdA KO* mutant cells phagocytosed as efficiently as WT cells. However, at increasing cell densities, *spdA KO* and *spdA-ins* mutant cells exhibited marked differences with WT cells: phagocytosis was inhibited more readily in *spdA KO* cells than in WT cells, and less in *spdA-ins* mutant cells than in WT cells. These results suggest that SpdA is involved in the pathway allowing cells to control their adhesion and phagocytosis as a response to cell density. SpdA may be directly involved in phagocytosis and cell spreading, and mobilized only when cells are growing at high densities and thus are exposed to high concentration of inhibitory quorum-sensing factors. Alternatively, SpdA may play a negative role in the quorum-sensing signaling pathway inhibiting cell adhesion and phagocytosis. Note that in *spdA* mutant cells, some phenomena implicating actin dynamics are affected (cell spreading, phagocytosis), while others are not (actin skeleton morphology, cell motility). It is thus possible that SpdA is involved specifically in certain facets of actin dynamics (cell spreading and phagocytosis) and not in others (e.g. cell motility).

As detailed above, s*pdA-ins* and *spdA KO* mutations generate opposite phenotypes: phagocytosis and cell spreading are increased in *spdA-ins* cells and decreased in *spdA KO* cells compared to WT cells. In both cases three independent clones were analyzed, suggesting strongly that the phenotypes observed are truly generated by the mutations. This result suggests that in *spdA-ins* mutant cells, SpdA is either stabilized or hyperactive. We generated anti-SpdA recombinant antibodies (MRB19, 20 and 21). While they readily detected a full-length GFP-SpdA protein, they failed to detect endogenous SpdA, and this prevented us from testing whether the amount of SpdA was actually increased in *spdA-ins* mutant cells (data not shown). Very little is known on how *Dictyostelium* cells modulate phagocytosis, adhesion and cell motility in response to secreted quorum-sensing factors. SpdA is the first identified protein that may participate in these regulatory pathways. Further studies will be necessary to establish the precise molecular mechanisms linking SpdA to the regulation of cell spreading and phagocytosis.

## Supporting Information

S1 Fig Generation of *spdA-ins* mutant cells. **A**nalysis of the genomic alteration of the original *SpdA-ins* mutant, design and usage of a mutagenic vector to create new *SpdA-ins* mutant cells.
(TIF)

S2 Fig Cell migration is unaffected in *spdA-ins* mutant cells. Analysis of random cell migration of a glass surface revealed no difference between WT and *SpdA-ins* mutant cells.
(TIF)

S3 Fig Generation of *spdA-ins* mutant cells. Design of a vector to create *SpdA KO cells*, and selection of the mutant clones.
(TIF)

S1 Table *Dictyostelium* gene products with significant homology to SpdA. (DOCX)
