# Supplementary figures and images for: Role of SpdA in Cell Spreading and Phagocytosis in Dictyostelium
Source: PLoS One. 2016 Aug 11;11(8):e0160376. doi: 10.1371/journal.pone.0160376 (PMC4981364; doi:10.1371/journal.pone.0160376)

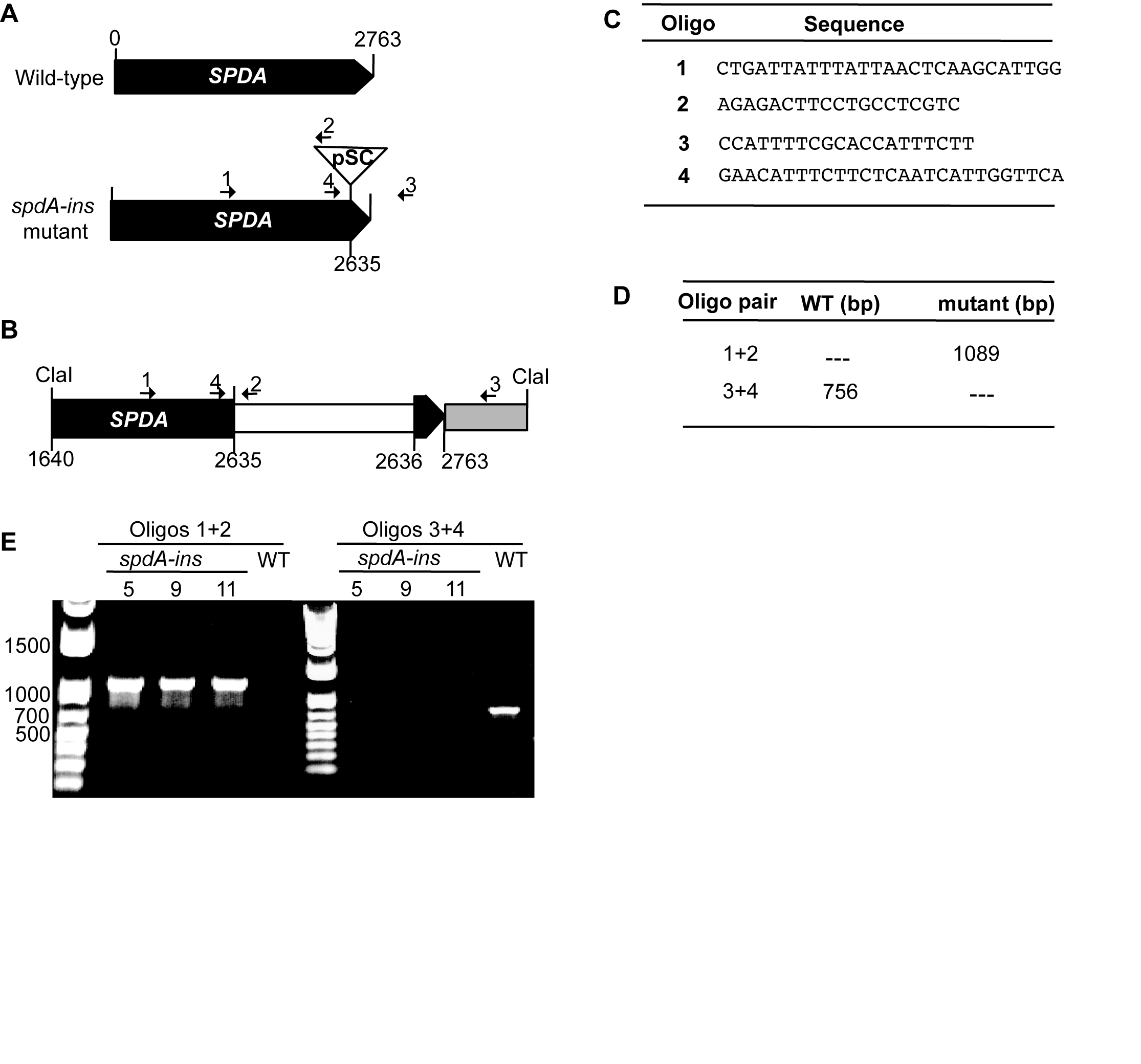

Supplement: S1 Fig — Analysis of the genomic alteration of the original SpdA-ins mutant, design and usage of a mutagenic vector to create new SpdA-ins mutant cells. (TIF) [file pone.0160376.s001.tif]

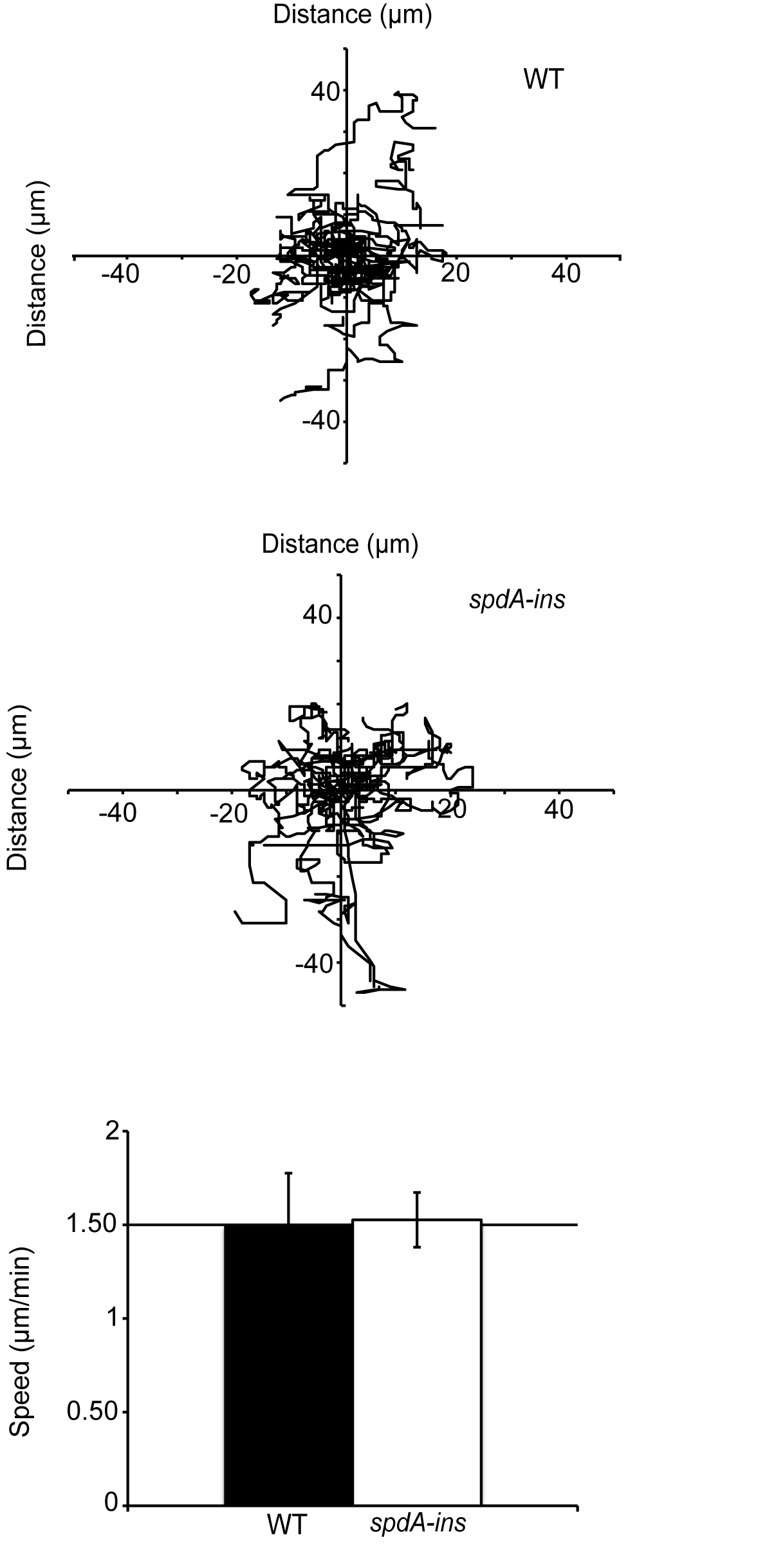

Supplement: S2 Fig — Analysis of random cell migration of a glass surface revealed no difference between WT and SpdA-ins mutant cells. (TIF) [file pone.0160376.s002.tif]

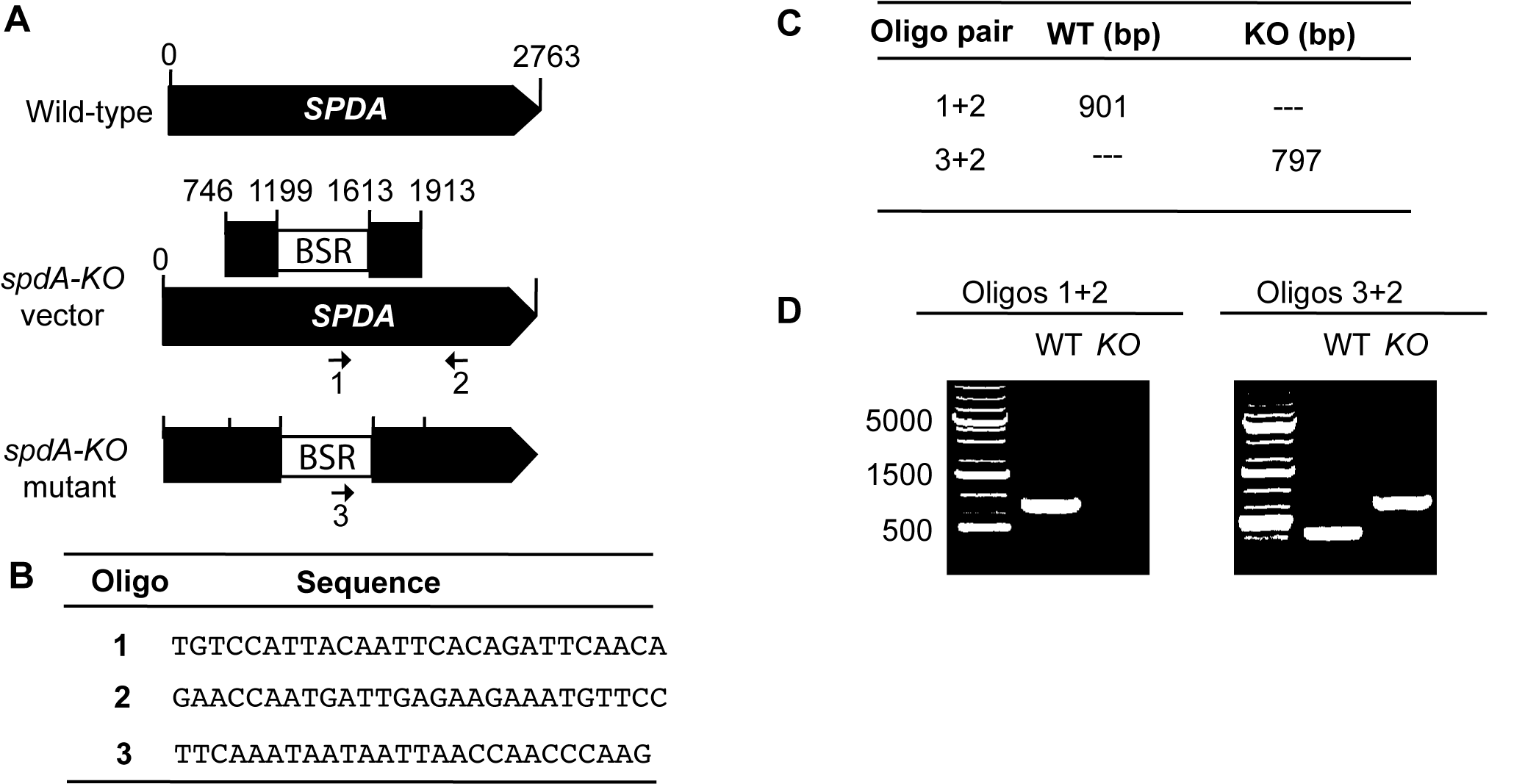

Supplement: S3 Fig — Design of a vector to create SpdA KO cells, and selection of the mutant clones. (TIF) [file pone.0160376.s003.tif]
