# Supplementary material for: Role of SpdA in Cell Spreading and Phagocytosis in Dictyostelium
Source: PLoS One. 2016 Aug 11;11(8):e0160376. doi: 10.1371/journal.pone.0160376 (PMC4981364; doi:10.1371/journal.pone.0160376)
Supplement: S1 Table — (DOCX) [file pone.0160376.s004.docx]

**S1 Table. *Dictyostelium* gene products with significant homology to SpdA (120 N-terminal residues).**

Proposed Gene ID Size (AA) chromosomal Id with

Name (DDB_) location SpdA

SpdA G0287845 920 5(809663-812425)

SpdB G0293782 763 6 (3259115-3261522) 45/114

SpdC G0293040 728 6 (2376674-2378860) 43/115

SpdD G0273953 675 2(3594233-3596260) 43/117

SpdE G0272903 675 2(2435526-2437553) 43/117

SpdF G0279845 832 3(2628470-2630968) 41/122

SpdG G0292932 768 6(2286339-2288645) 38/109

SpdH G0293744 845 6(3261758-3264389) 44/148

SpdI G0280509 884 3(3464216-3466870) 43/144

SpdJ G0287615 782 5(470940-473288) 29/98

SpdK G0272058 883 2(1124620-1127271) 29/56

SpdL G0292950 774 6(2289490-2291814) 37/115

SpdM G0278247 888 3(552139-554805) 27/55

SpdN G0292952 908 6(2292229-2294955) 24/60

SpdO G0292936 819 6(2298560-2301019) 38/117

SpdP G0268692 325 1(1928290-1929267) 26/62

SpdQ G0292934 780 6(2295609-2297951) 22/54

SpdR G0292990 679 6(2303391-2305430) 26/59

SpdS G0267746 535 1(774330-775937) 26/64
